# Characterization of *Solanum melongena* Thioesterases Related to Tomato Methylketone Synthase 2

**DOI:** 10.3390/genes10070549

**Published:** 2019-07-18

**Authors:** Vy Le Uyen Khuat, Vi Thi Tuong Bui, Huong Thi Diem Tran, Nuong Xuan Truong, Thien Chi Nguyen, Phuc Huynh Hanh Mai, Tuan Le Anh Dang, Hiep Minh Dinh, Hong Thi Anh Pham, Thuong Thi Hong Nguyen

**Affiliations:** 1 Faculty of Biology and Biotechnology, University of Science, Vietnam National University, Ho Chi Minh City 700000, Vietnam; 2 VN-UK Institute for Research and Executive Education, University of Danang, Danang City 550000, Vietnam; 3 Agricultural Hi-tech Park, Ho Chi Minh City 700000, Vietnam

**Keywords:** β-ketoacids, 2-methylketones, methylketone synthase 2, methyl jasmonate, methyl salicylate, *Solanum melongena*, thioesterase

## Abstract

2-Methylketones are involved in plant defense and fragrance and have industrial applications as flavor additives and for biofuel production. We isolated three genes from the crop plant *Solanum melongena* (eggplant) and investigated these as candidates for methylketone production. The wild tomato methylketone synthase 2 (ShMKS2), which hydrolyzes β-ketoacyl-acyl carrier proteins (ACP) to release β-ketoacids in the penultimate step of methylketone synthesis, was used as a query to identify three homologs from *S. melongena*: SmMKS2-1, SmMKS2-2, and SmMKS2-3. Expression and functional characterization of SmMKS2s in *E. coli* showed that SmMKS2-1 and SmMKS2-2 exhibited the thioesterase activity against different β-ketoacyl-ACP substrates to generate the corresponding saturated and unsaturated β-ketoacids, which can undergo decarboxylation to form their respective 2-methylketone products, whereas SmMKS2-3 showed no activity. *SmMKS2-1* was expressed at high level in leaves, stems, roots, flowers, and fruits, whereas expression of *SmMKS2-2* and *SmMKS2-3* was mainly in flowers and fruits, respectively. Expression of *SmMKS2-1* was induced in leaves by mechanical wounding, and by methyl jasmonate or methyl salicylate, but *SmMKS2-2* and *SmMKS2-3* genes were not induced. SmMKS2-1 is a candidate for methylketone-based defense in eggplant, and both SmMKS2-1 and SmMKS2-2 are novel MKS2 enzymes for biosynthesis of methylketones as feedstocks to biofuel production.

## 1. Introduction

Methylketones are plant metabolites that have strong insecticidal activity against a wide range of anthropod insects [1] and are also assumed to contribute to the fragrance and flavor of fruits [2,3]. Recently, methylketones have also received interest as potential feedstocks to biofuel production [4,5]. The carbon skeletons for the synthesis of methylketones come from the fatty acid biosynthetic pathway. Until now, two methylketone synthase enzymes involved in methylketone biosynthesis, namely methylketone synthase 2 (MKS2) and methylketone synthase 1 (MKS1), have been functionally characterized in wild tomato species *Solanum habrochaites* subsp. *glabratum* [6]. Since the first functional characterization of the wild tomato *Solanum habrochaites* methylketone synthase 2 (ShMKS2) as a β-ketoacyl-acyl carrier proteins (ACP) thioesterase that catalyzes the penultimate step of the methylketone biosynthetic pathway, homologs of ShMKS2 have been isolated in the cultivated tomato *Solanum lycopersicum* and in the model plant *Arabidopsis thaliana*. All of them could also hydrolyze fatty acyl-ACP and/or β-ketoacyl-ACP substrates to release fatty acids or β-ketoacids, respectively [6,7]. β-Ketoacids are unstable and can readily undergo the non-enzymatic elimination of carbon dioxide under some conditions to form 2-methylketone decarboxylated products [8]. Decarboxylation of β-ketoacids could also be accelerated by an enzyme possessing decarboxylase activity such as methylketone synthase 1 (MKS1) present in the *S. habrochaites* glands. Methylketones are found in large quantities in the trichomes of this wild tomato species but are present at low levels in the cultivated tomato. Little is known about the methylketone accumulation, and the enzymes involved, in other Solanaceae species. 

Interestingly, those plant-derived MKS2s, also known as acyl-lipid thioesterases (ALTs), generated different fatty acid and β-ketoacid profiles when recombinantly expressed in *E. coli* [6,7,9]. None of them shared the same catalytic profile in *E. coli*. Several ALT-like enzymes produced the same primary products but different minor products, others generated the same types but different ratios of products. In view of such broad catalytic diversity within the characterized MKS2/ALTs, screening for additional MKS2/ALT-like thioesterases from a wide range of plant species will likely diversify the enzyme collection and provide us with valuable input to gain insight into how the catalytic divergence happened in this enzyme family.

The Solanaceae family includes many species that have important roles in agriculture and medicine. Genome sequences of several species in this family have been determined for studies at molecular and cellular levels such as those of tomato (*Solanum lycopersicum*) [10], potato (*Solanum tuberosum*) [11], eggplant (*Solanum melongena*) [12], pepper (*Capsicum annuum*) [13,14], and tobacco (*Nicotiana tabacum*) [15]. Eggplant is one of the most popular vegetables in Asia, the Middle East, Southern Europe and Africa [16]. While the genetics of many agronomically important traits of tomato and potato has been extensively studied, the same is not true for the eggplant. It is noteworthy that eggplant (*S. melongena* L.) is a representative of the Leptostemonum clade, the largest monophyletic group in the Solanaceae family. Eggplant rose in Africa and were dispersed throughout the Middle East to Asia, while most important plants in Solanaceae, such as tomatoes, potatoes, peppers and tobacco originated in the Americas [17]. The accumulation of genetic information of eggplant thus will be valuable for comparative biological studies of genetics, physiology, development, and evolution of the Solanaceae family. Recently, the eggplant genome was substantially determined [12], and a draft genome sequence of *S. melongena* was built and uploaded on https://solgenomics.net/. In addition, the Eggplant Genome Consortium has released a pre-publication version of their eggplant genome on http://www.eggplantgenome.org. These would be an excellent source for prospecting novel genes in this species. 

We initiated a study of 2-methylketone synthesis in *S. melongena* L. to investigate the contribution of these compounds in insect resistance, fragrance and flavor in the crop plant, and also a possible biofuel blending agents. Here, we report the partial characterization of three thioesterases from *S. melongena* L. that are related to tomato methylketone synthase 2.

## 2. Materials and Methods 

### 2.1. Plant Growth and Treatment Conditions

Seeds of eggplant (*Solanum melongena* L.) were obtained from the Southern Fruit Research Institute (SOFRI), Vietnam.

For the isolation of full-length cDNAs of eggplant *MKS2* genes and determination of tissue specific gene expression, 13-week-old eggplant plants were dissected to obtain material of each of the following tissues: young leaves, mature leaves, roots, stems, flowers, and fruits. 

For stress treatments, plants were grown in a greenhouse under the same conditions of temperature and light. The six-week-old plants were used for different stress treatments. Wounding was performed by crushing eggplant leaves two times across the midvein with a hemostat, and leaves were collected for analysis after 24 h [18]. For the methyl jasmonate (MeJA) treatment, each intact plant was enclosed in a 4-L airtight glass container with one 1-cm^2^ piece of filter paper carrying 6.6 µL (or 6.8 mg) of MeJA. All the plants exposed to MeJA vapor for 2 h were further grown in the greenhouse for 24 h before their leaves were collected for gene expression analysis [19]. Methyl salicylate (MeSA) treatment was conducted using similar experimental setting of the MeJA treatment experiment, in which 2.53 µL (or 3 mg) of MeSA was applied onto a 1-cm^2^ piece of filter paper [20]. Untreated plants were used as control for each experiment. All experiments were repeated three times, each time with three biological replicates. Leaves were sampled and immediately frozen in liquid nitrogen and stored at −80 °C.

### 2.2. Identification of *SmMKS2* Genes in the Eggplant Genome 

The amino acid sequence of ShMKS2 protein (Genbank accession: ADK38536.1) was used as a query in a translated basic local alignment search tool (TBLASTN) search against the Eggplant Genome Database (http://eggplant.kazusa.or.jp) to identify scaffolds that contain the MKS2 homologs, designated as SmMKS2s. The FGENESH gene finder program (http://www.softbery.com) [21] was used for initial annotation of the predicted eggplant *MKS2* genes. 

### 2.3. cDNA Isolation and Phylogenetic Analysis of *SmMKS2* Sequences

The total RNA was isolated from the collected tissues and converted to cDNA using ProtoScript^®^II First Strand cDNA Synthesis kit (New England Biolabs, Massachusetts, USA) and oligo (dT)_18_ primer. Full-length cDNA sequences for *SmMKS2s* were obtained by polymerase chain reaction (PCR) using 1 μL of the resulting cDNA and gene-specific primers (Supplementary File 8). The PCR products were purified using Expin™ Gel SV (GeneAll Biotechnology Co., Ltd., Seoul, Korea) and cloned into pGEM-T Easy cloning vector (Promega, Wisconsin, USA). The recombinant plasmids were transformed into *E. coli* TOP10 competent cells. Positive clones were selected by blue/white colony screening and PCR colony. Recombinant plasmids were extracted using StrataPrep Plasmid Miniprep Kit (Agilent Technologies, California, USA) and their inserts were sequenced. Three SmMKS2 sequences have been deposited in GenBank with accession number MK990608, MK990609, and MK990610, respectively. Phylogenetic and molecular evolutionary analyses were conducted using MEGA X [22]. 

### 2.4. Gene Expression Analysis by Quantitative Real-Time Polymerase Chain Reaction (qRT-PCR)

Total RNA was isolated from the described tissues using the Ribospin™ Plant kit (GeneAll Biotechnology Co., Ltd., Seoul, Korea) according to the manufacturer’s instructions. The isolated RNA was treated with RNase-free DNase I (Thermo Scientific, Massachusetts, USA) to remove genomic DNA contamination. The integrity of the extracted RNA was assessed by agarose gel electrophoresis. One and a half µg of DNA-free RNA was reverse transcribed using ProtoScript^®^ II First Strand cDNA Synthesis (New England Biolabs, Massachusetts, USA) and oligo (dT)_18_ primer. Gene-specific primers were designed using OligoAnalyzer 3.1 (Integrated DNA Technologies, Inc., Iowa, USA) and Primer-BLAST program provided by the National Center for Biotechnology Information (NCBI) (Supplementary File 8). Quantitative real-time PCR was performed with CFX96 Touch Real-Time PCR Detection System (Bio-Rad Laboratories, Inc., California, USA) using SolGent *h-Taq* DNA Polymerase (SolGent, Daejeon, Korea) and EvaGreen DNA-binding Dye (Biotium, California, USA) according to the manufacturer’s instructions. The cDNA was diluted from 1:5 to 1:100, and 2 µL was subjected to each quantitative real-time polymerase chain reaction (qRT-PCR) in a final volume of 12.5 µL containing 1X SolGent *h-Taq* Reaction Buffer, 0.5X EvaGreen, 2.5 mM MgCl_2_, 0.2 mM dNTP, 0.3 U *h-Taq* polymerase, 400 nM each for forward and reverse primers. The amplification parameters were one cycle at 95 °C for 15 min, 40 cycles of 95 °C for 20 s, 55 °C for 40 s, and 72 °C for 7 s. Transcript levels of *SmMKS2-1*, *SmMKS2-2* and *SmMKS2-3* were analyzed by qRT-PCR using gene-specific primers (Supplementary File 1), normalized to the eggplant reference gene adenine phosphoribosyl transferase (APRT) (Genbank accession: JX448345.1) and expressed as relative expression [23]. Each data point represents an average of at least three independent biological samples with three technical replicates for each sample.

### 2.5. Expression of *SmMKS2-1*, *SmMKS2-2* and *SmMKS2-3* in *E. coli*

The coding regions of *SmMKS2-1*, *SmMKS2-2*, and *SmMKS2-3* genes (minus the transit peptide encoding region) were amplified by Phusion High Fidelity DNA Polymerase (Thermo Scientific, Massachusetts, USA) to add *Nde*I and *Xho*I restriction sites, spliced into pETDuet-1 (Invitrogen, California, USA), and transformed into *E. coli* strain C41(DE3) (Invitrogen). The *E. coli* C41(DE3) cells containing the plasmid pETDuet-1-SmMKS2-x (x = 1/2/3) was grown in Terrific Broth (TB) medium containing 100 μg/mL ampicillin until an OD_600nm_ reached 0.6 to 0.8. Expression of the recombinant protein was induced by addition of 0.5 mM isopropylthio-β-galactoside (IPTG) and the culture was kept at 18 °C for 16 h. The cell cultures were collected for further analysis. A negative control experiment (*E. coli* C41(DE3) cells carrying the empty plasmid pETDuet-1) was performed in parallel.

For protein expression analysis, bacterial cells were harvested by centrifugation (6000 × *g*, 4 °C, 10 min), and resuspended in the lysis buffer (100 mM NaCl; 10 mM Tris; 1 mM EDTA; 10% glycerol; pH 8). Cells were lysed by sonication and the total cell lysate was centrifuged (22,000× *g*, 4 °C, 3 min) to pellet the cellular debris: the lysis supernatant was collected as the soluble protein fraction, and the pellet as the insoluble fraction. The pellet was then re-suspended in an equal volume of the lysis buffer. Samples of total cell lysate, soluble and insoluble protein fractions were boiled for 10 min at 98 °C in a reducing loading buffer (0.1 M Tris-HCl, pH 6.8, 1% sodium dodecyl sulfate (SDS), 10% glycerol, 0.003% bromophenol blue, and 1% β-mercaptoethanol) [24] to denature proteins before they were loaded on the gel. Prepared samples were run through 4% stacking gel and 16% separating gel in Tris-Tricine-SDS Buffer 1X (0.1 M Tris, 0.1 M Tricine and 0.1% SDS, pH 8.25) [25] at a constant voltage of 90 V. The gel was stained with Coomassie Blue. 

For methylketone profile analysis, hexane was added to the cell culture of *E. coli* expressing SmMKS2 and the mixture was then incubated at 75 °C for 30 min to convert β-ketoacids into the corresponding 2-methylketones. After the extraction, the mixture was cooled at 18 °C for 10 min, vortexed and centrifuged at 5,000 rpm for 10 min. The gas chromatography–mass spectrometry (GC–MS) analysis was conducted by injecting 1 μL of the resulting extract into an HP5 MS column (30 m × 0.25 mm × 0.25 µm) installed on a gas chromatograph (Agilent 6890N) coupled to an Agilent 5972 Mass Spectrum Detector. Helium was used as the carrier gas at a constant flow rate of 1 mL/min. The MS was operated in SCAN mode and molecular ions were typically scanned from 40 to 500 atomic mass units. The injector was programmed to hold at 250 °C for 0.5 min, ramp to 280 °C at 50 °C/min, and hold for 1 min. The column oven was programmed to initialize at 60 °C, hold for 5 min, then ramp to 300 °C at a rate of 15 °C/min. For saturated methylketones, peaks identified as 2-heptanone, 2-nonanone, 2-undecanone, and 2-tridecanone by the similarity search in NIST 14 MS Search program were further confirmed by comparing their retention times with those of 2-methylketone standards (Sigma-Aldrich). For monounsaturated ketones, as no authentic standards were available, peaks were identified based on their previously reported mass spectrum [4,7]. Quantification of methylketones was performed as previously described [6]. 

### 2.6. Accession Numbers

The GenBank accession numbers for the sequences referred to in this paper are given in parentheses: *SmMKS2-1* (MK990608), *SmMKS2-2* (MK990609) and *SmMKS2-3* (MK990610).

### 2.7. Data Analysis

In order to study the effect of wounding, MeJA, and MeSA on the transcriptional expression of *SmMKS2* genes, we first used one-way analysis of variance (ANOVA) and then assessed significant difference between treated plants and control plants by Dunnett’s post hoc comparisons.

## 3. Results

### 3.1. Identification of *MKS2* Genes in *S. melongena*

TBLASTN searches with the known wild tomato ShMKS2 protein sequence against the Eggplant Genome Database identified three scaffolds (Sme2.5_06004.1, Sme2.5_20884.1, and Sme2.5_02928.1) with an e-value lower than 2 × 10^−52^, each of which contained DNA fragments encoding amino acid sequences with significant similarity to different subregions of the ShMKS2 protein. Three putative open reading frames with high identity to ShMKS2 were predicted from the identified scaffolds, and designated as *SmMKS2-1*, *SmMKS2-2*, and *SmMKS2-3*, respectively. 

Isolation of cDNA and genomic sequences corresponding to *SmMKS2* genes was performed by PCR using primers that were designed based on the predicted open reading frames (ORFs). Subsequent sequencing and alignment of the cDNA sequence to genomic sequence revealed that *SmMKS2-1* spanned approximately 4364 bp on eggplant scaffold Sme2.5_06004.1 and consisted of five exons. The complete coding region of *SmMKS2-1* gene is 618 bp long and encodes a 205 amino acid protein (23.4 kDa) (Supplementary Files 1 and 2). 

While the released draft eggplant genome sequence suggested that *SmMKS2-2* is present in the scaffold Sme2.5_20884.1, the incomplete and erroneous assembly of this scaffold is likely the cause of our initial failure in isolating the full-length cDNA sequence of *SmMKS2-2* using primers based on the predicted *SmMKS2-2* ORF. Initially, the longest cDNA of *SmMKS2-2* we could obtain was 426 bp long and the first ATG codon of the open reading frame in this cDNA was equivalent to the ATG codon that occurs in positions 2 to 4 of exon 2 in the *SmMKS2-1* genes (Supplementary Files 2 and 3). In order to determine the beginning of the transcript of *SmMKS2-2*, we performed TBLASTN searches against Transcriptome Shortgun Assembly (TSA) database of Solanum (taxid:4107) using the amino acid sequence deduced from the 426-bp cDNA segment of *SmMKS2-2* as a query and identified a homologous sequence from *S. prinophyllum* (accession: GEZT01051720.1) with the highest amino acid sequence identity (91%). This sequence shared 100% identity with the first 159 residues of the query sequence and thus was utilized as a framework for designing forward primer to retrieve the 5′-end of *SmMKS2-2* cDNA. A PCR experiment was performed using the above-designed forward primer and a reverse primer complementary to the 3′ end of the coding region. Analysis of the DNA fragments produced in this experiment by agarose gel electrophoresis gave a sharp band (Supplementary File 1). Subsequent sequencing of the resulting fragment indicated that the *SmMKS2-2* transcript was 180 nucleotides longer at its 5′ ends than the initially isolated cDNA. As a result, the full-length cDNA sequence of *SmMKS2-2* obtained from eggplant flower tissue was 606 bp long and encodes for a 22.5-kDa protein of 201 amino acids. The newly uncovered 5′ end sequence was homologous to exon 1 in the *SmMKS2-1* gene, which encodes a putative N-terminal transit peptide as seen in all other known homologous genes from *Arabidopsis* and tomato. The complete genomic sequence of *SmMKS2-2* gene was determined and shown in Supplementary File 3. The *SmMKS2-2* gene is 3424 bp long and has five exons and four introns. 

Amplification of the *SmMKS2-3* cDNA by RT-PCR using primers that are complementary to the 5′ and 3′ end of the predicted full-length ORFs on the eggplant scaffold Sme2.5_02928.1 gave a sharp band (Supplementary File 1). Sequencing the resulting fragment showed that *SmMKS2-3* contains an in-frame stop codon in exon 4 in addition to a 47-nucleotide insertion at the end of the third exon that is predicted to cause a frameshift. *SmMKS2-3* has a GT to GC transition at nucleotides 40β-404 (this dinucleotide corresponds to the 5′ splice site of intron 3 in *SmMKS2-1* and *SmMKS2-2*) and as a result, the new 5′ splice site shifted 47 nucleotides to the right in the *SmMKS2-3* sequence compared to *SmMKS2-1* and *SmMKS2-2* sequences. The insertion of 47 intronic nucleotides into the SmMKS2-3 transcript results in a frameshift that leads to a premature stop codon within the fourth exon (Supplementary File 4). Thus, the *SmMKS2-3* gene has only four exons and three introns and encodes a smaller protein of 156 amino acids (17.1 kDa).

### 3.2. Phylogenetic Analysis of the Eggplant SmMKS2s

**Figure 1:**
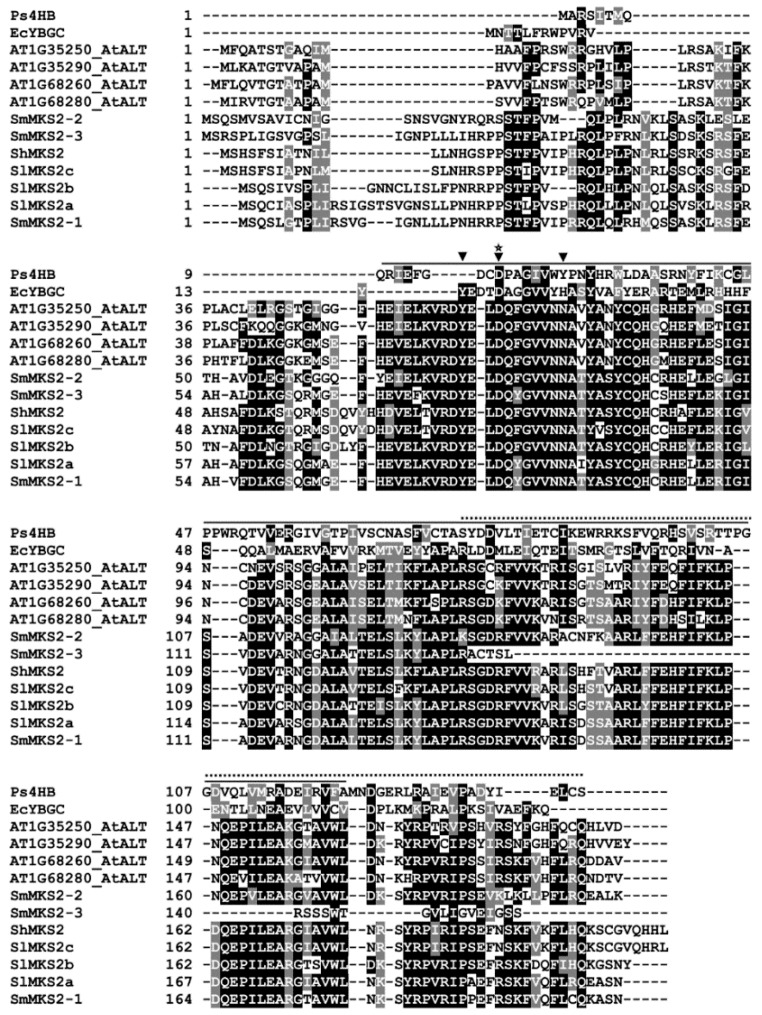
Amino acid sequence alignment of SmMKS2 with functionally characterized methylketone synthase 2/acyl-lipid thioesterases (MKS2/ALTs). The deduced amino acid sequences of *SmMKS2-1*, *SmMKS2-2*, and *SmMKS2-3* are compared to previously reported *At1g68260* (AEE34774.1), *At1g68280* (AEE34776.2), *At1g35290* (AEE31776.1), and *At1g35250* (AEE31773.1) from *A. thaliana*, *ShMKS2* (ADK38536.1) from the wild tomato *Solanum habrochaites*, *SlMKS2a* (ADK38541.1), *SlMKS2b* (ADK38542.1) and *SlMKS2c* (ADK38543.1) from the cultivated tomato *Solanum lycopersicum*, *Ps4HB* (EF569604) from *Pseudomona*, and *EcYbgC* from *Escherichia coli.* Identical residues are highlighted in black and similar residues are highlighted in grey. The arrowheads indicate positions of catalytic residues defined in members of the thioesterase family 9, including the structurally resolved *EcYbgC* from *E. coli*. The conserved Asp residue required for thioester bond cleavage is marked with star. Black horizontal line indicates the conserved Hotdog fold region present in MKS2/ALTs, and the dashed horizontal line indicates C-terminal residues lost in *SmMKS2-3* and replaced by new amino acids encoded by 47 *intronic* nucleotides plus the first nucleotide of exon 4.

A multiple sequence alignment of the SmMKS2 sequences and a subset of the homologous proteins showed that the eggplant sequences included a conserved aspartate (Asp) required for the catalytic activity of single Hotdog fold thioesterases (Figure 1). The Asp residue was located at position 17 in the 4-hydroxybenzoyl-CoA thioesterase (4HBT) from *Pseudomonas* sp. strain CBS and previously identified as the catalytic residue required for thioester bond cleavage [26]. In line with this, conversion of the equivalent aspartate to alanine abolished the thioesterase activity of ShMKS2 and ALT1 [6,7]. In addition, the full-length amino acid sequence of SmMKS2-1 and SmMKS2-2 also contained a C-terminal Hotdog-fold superfamily domain, a typical domain of single Hotdog fold acyl-ACP thioesterases found in different plant taxa [6,7]. Based on the amino acid sequence, thioesterases have been divided into 23 different families and the plant MKS2 proteins belong to the thioesterase family 9 (TE9) [27]. Structural characterization of YbgC thioesterases in TE9 [27,28] and the alignment of the plant MKS2 sequences with EcYbgC (Figure 1) identified Tyr^78^, Asp^81^ and Asn^88^ (numbered relative to the sequence of SmMKS2-1) as the most likely candidates for catalytic triad residues of the plant MKS2s. Notably, SmMKS2-3 also contained the putative catalytic triad, but lost 37 residues in the C-terminal region of Hotdog domain because of the presence of a premature termination codon (Figure 1).

**Figure 2:**
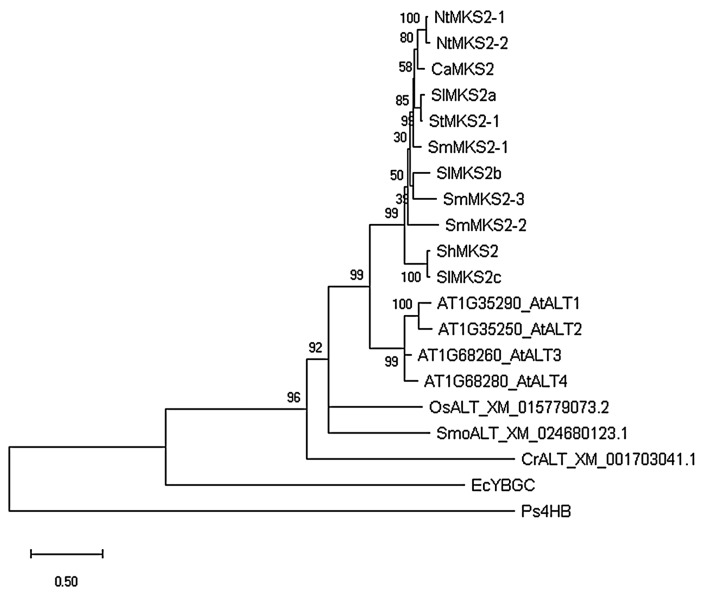
Phylogenetic analysis of *SmMKS2* genes. Maximum likelihood phylogenetic tree of the Solanaceae *MKS2*s and previously reported *ALT*s from *A. thaliana* and Ps4HB (EF569604) from *Pseudomonas*. Bootstrap values were performed with 1000 replicates and indicated next to the branches. At, *Arabidopsis thaliana*; Ca, *Capsicum annuum*; Nt, *Nicotiana tabacum*; Sh, *S. habrochaites*; Sl, *S. lycopersicum*; St, *S. tuberosum*; Os, *Oryza sativa*; Smo, *Selaginella moellendorffii*; Cr, *Chlamydomonas reinhardtii*; Ec, *E. coli*; Ps, *Pseudomonas*.

To investigate the phylogenetic relationship of *SmMKS2* genes with single Hotdog fold thioesterase genes found in other species, a phylogenetic analysis based on the coding sequences of MKS2s from several Solanaceace plant species (*Solanum melongena*, *Solanum habrochaites*, *Solanum lycopersicum*, *Nicotiana tabacum*, *Capsicum annuum*, *Solanum tuberosum*), as well as representative MKS2 sequences from bacteria (*E. coli* and *Pseudomonas*), algae *(Chlamydomonas reinhardtii*), lycophytes (*Selaginella moellendorffii*), monocots (*Oryza sativa*), and dicots (*Arabidopsis thaliana*) was built using the maximum likelihood method. The phylogenetic tree makes clear that all the Solanaceae *MKS2* genes form a distinct clade that diverged from *AtALT* genes. *ShMKS2* and *SlMKS2c* form a separate branch, and all *SmMKS2* genes are located on a parallel branch together with other Solanaceae *MKS2* genes. *SmMKS2-2* appears to be distinct but closely related to other Solanaceae MKS2 genes (Figure 2). A second analysis of these genes based on Bayesian inference was also conducted (Supplementary File 5), and it generated essentially the same phylogenetic tree, but with much higher bootstrap values to some branch points in the clade included *MKS2* genes from the Solanaceae species.

### 3.3. *SmMKS2* Genes are Differently Expressed in Various Eggplant Organs

**Figure 3:**
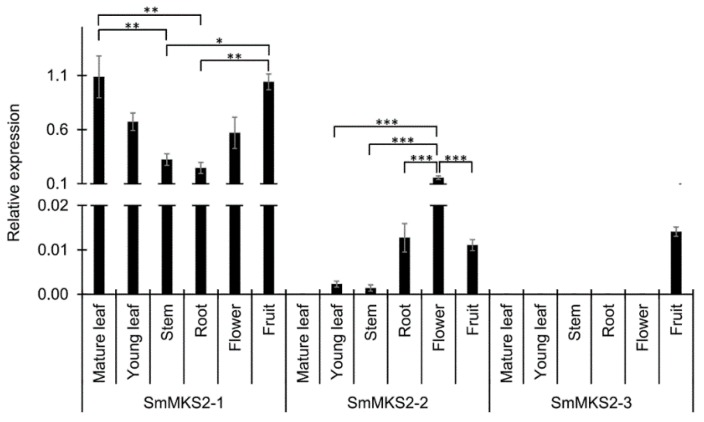
Spatial expression patterns of three *SmMKS2* genes in eggplant determined by quantitative real-time polymerase chain reaction (qRT-PCR) analysis. Transcript levels were normalized with respect to the adenine phosphoribosyl transferase gene (*APRT*, Genbank accession: JX448345.1). Values are mean ± standard error (SE) from three replicates. Significant differences were assessed by one-way analysis of variance (ANOVA) followed by Tukey’s test: * *p* < 0.01, ** *p* < 0.005, *** *p* < 0.001.

The mRNA expression of *SmMKS2* in different eggplant organs was investigated by quantitative real-time polymerase chain reaction (qRT-PCR) analysis. Transcripts of *SmMKS2-1* were detected in multiple plant tissues, both aerial and root parts, but were highest in leaves and fruits. The highest level of *SmMKS2-2* transcripts was in flowers, with much lower but detectable levels in all other tissues tested. The expression pattern of *SmMKS2-3* transcripts were detected only at very low levels in fruits. In the plant, the magnitude of *SmMKS2-2* and *SmMKS2-3* expression was much lower than that of *SmMKS2-1* (Figure 3).

### 3.4. SmMKS2-1 but Neither SmMKS2-2 Nor SmMKS2-3 is Induced by Artificial Wounding, Methyl Jasmonate, and Methyl Salicylate

**Figure 4:**
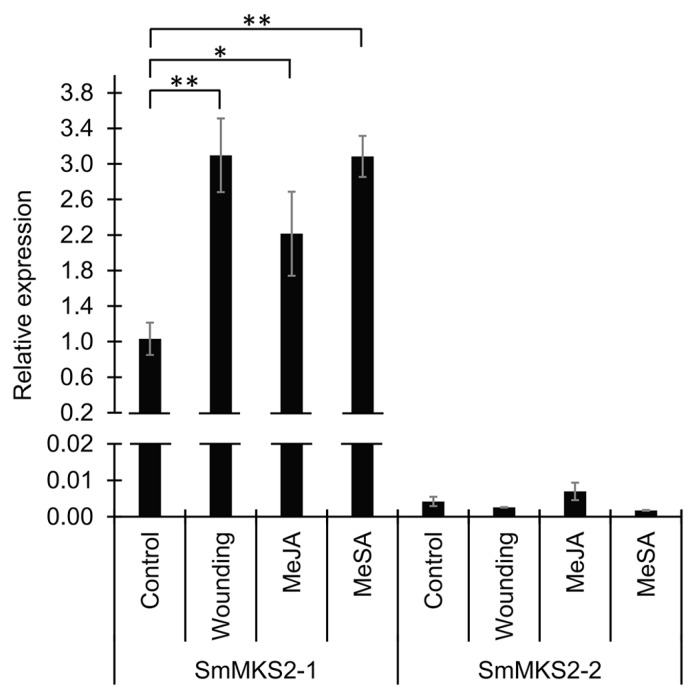
Expression profiles of *SmMKS2-1* and *SmMKS2-2* genes in response to wounding, MeJA and MeSA treatment. Values are means ± SE from three replicates. Significant differences were assessed by a Dunnett’s test: * *p* < 0.05, ** *p* < 0.01.

To assess whether *SmMKS2* gene*s* are involved in the regulation of stress responses in *S. melongena*, changes in their transcriptional expression in eggplant leaves upon artificial wounding, and exogenous application of MeJA and MeSA were analyzed by qRT-PCR (Figure 4). Transcriptional expression of *SmMKS2-1* was indeed induced in leaves by these stress factors. Expression of *SmMKS2-2* was not induced in leaves following mechanical wounding, or treatment with MeJA or MeSA for 24 h. *SmMKS2-3*, which was undetectable in leaves (Figure 3), was not activated after treatment. Taken together, these results suggest that *SmMKS2-1* might play a role in stress responses via MeJA- and MeSA-mediated signaling pathways in *S. melongena*.

### 3.5. Expression of *SmMKS2* Genes in *E. coli* Led to the Production of β-Ketoacids

The expression of SmMKS2 proteins in *E. coli* C41(DE3) strain was verified by sodium dodecyl sulfate polyacrylamide gel electrophoresis (SDS-PAGE) gel analysis (Supplementary File 6). The SmMKS2 proteins were predominantly present in the soluble fractions prepared from *E. coli* cells harboring the recombinant plasmids but not in those transformed with the empty vector. Introduction of a premature termination codon into the fourth exon of the *SmMKS2-3* gene leads to premature termination of translation and thus, the molecular weight of SmMKS2-3 appears to be lower than that of SmMKS2-1, or SmMKS2-2 in SDS-PAGE (Supplementary File 6).

Putative intermediates in the synthesis of the final methylketone products are β-ketoacids. The unstable nature of β-ketoacids provides considerable challenges to the effort to detect and quantify them directly. However, β-ketoacids could be decarboxylated by heat treatment or by enzymatic catalysis to form the corresponding 2-methylketones, which can be measured by gas chromatography–mass spectrometry [6]. Thus, in order to test whether expression of each SmMKS2 (without its transit peptide) in the *E. coli* C41(DE3) strain resulted in the formation of β-ketoacids, we collected cell culture of bacterial cells expressing each of them, and analysed for the presence of final methylketone products formed after the culture was treated with heat (75 °C) for 30 min. 

**Figure 5:**
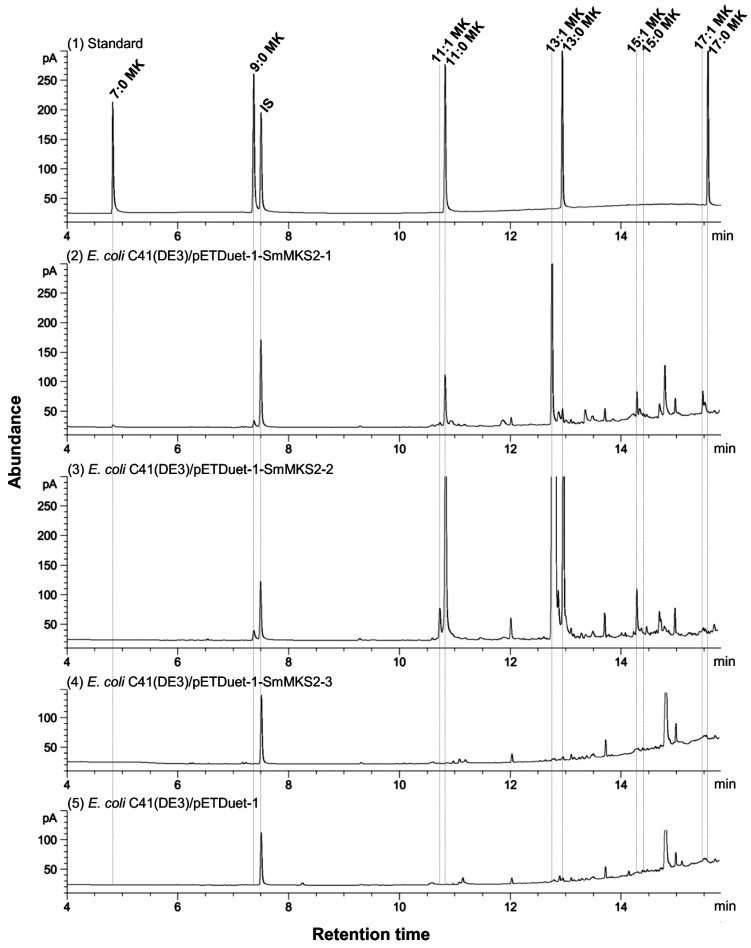
Identification of methylketone products from cell culture of *E. coli* C41(DE3) expressing three SmMKS2s by gas chromatography–mass spectrometry (GC–MS). 2-Methylketones were identified by GC–MS (see Supplementary File 7), and by comparing their retention times with those of 2-methylketone standards. (1) Authentic methylketone; (2) Cell culture of *E. coli* C41(DE3) expressing SmMKS2-1; (3) Cell culture of *E. coli* C41(DE3) expressing SmMKS2-2; (4) Cell culture of *E. coli* C41(DE3) expressing SmMKS2-3; (5) Cell culture of *E. coli* C41(DE3) carrying the empty vector (pETDuet-1).

**Figure 6:**
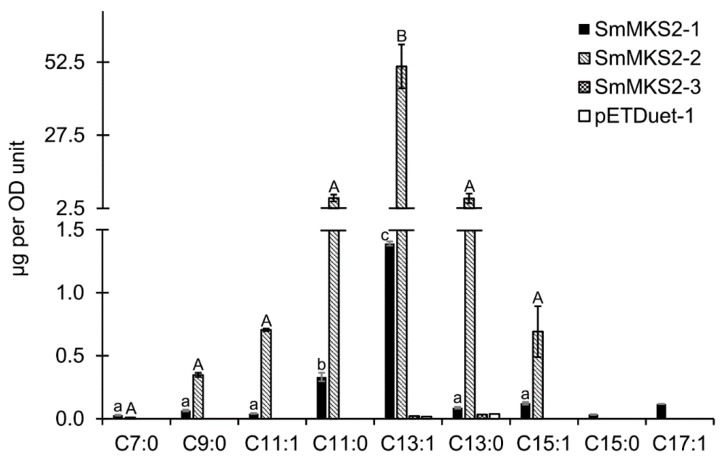
Quantification of 2-methylketones detected following heating the culture of *E. coli* cells *E. coli* cells carrying an empty vector, or expressing SmMKS2-1, SmMKS2-2, SmMKS2-3. Cells were grown and media were collected and treated as described in “Materials and Methods.” 2-Methylketones, which were derived from β-ketoacids, were quantified by comparison of peak areas to a linalool internal standard using GC. Values are averages ± SE calculated from three replicates. Significant differences were assessed by one-way ANOVA followed by Tukey’s test. Bars of the same pattern with different letters are significantly different (*p* < 0.005).

The culture of *E. coli* C41(DE3) cells expressing SmMKS2-1 released several types of odd-chain methylketones ranging from 7 to 17 carbons, including saturated methylketones such as 2-heptanone (7:0), 2-nonanone (9:0), 2-undecanone (11:0), 2-tridecanone (13:0), and 2-pentadecanone (15:0) and monosaturated methylketones such as (Z)-6-tridecen-2-one (13:1), (Z)-8-pentadecen-2-one (15:1), and putative 2-heptadecenone (17:1). Among them, (Z)-6-tridecen-2-one (13:1) was the most abundant product with 1391 ng/OD unit (Figure 5; Figure 6). SmMKS2-2 expression generated high levels of unsaturated medium-chain methylketone 13:1 (51,054 ng/OD unit) along with low levels of 2-undecanone (11:0) (6019 ng/OD unit) and 2-tridecanone (13:0) (5908 ng/OD unit), and even much lower levels of 2-nonanone (9:0) (348 ng/OD unit), (Z)-4-undecen-2-one (11:1) (705 ng/OD unit), and (Z)-8-pentadecen-2-one (15:1) (ng/mL). Levels of 13:1 methylketone produced by SmMKS2-2 were about 40 times higher than those produced by SmMKS2-1 (Figure 5 and Figure 6). The structures of saturated methylketone products were confirmed by the similarity search in NIST 14 MS Search program and by comparing their retention times with those of 2-methylketone standards. Given no authentic monounsaturated 2-methylketones standards were available, (Z)-4-undecen-2-one (11:1), (Z)-6-tridecen-2-one (13:1), and (Z)-8-pentadecen-2-one (15:1) were identified based on their previously reported mass spectrum [4,7]. Even though no mass spectra were available for 2-heptadecenone (17:1), the compound eluted at 15 min (Figure 5) shows a highly similar mass spectral fragmentation pattern to other annotated monosaturated methylketones and had molecular ion peak of m/z 252 corresponding to the relative molecular mass of 2-heptadecenone. As a negative control, culture of cells just carrying the same vector (pETDuet-1) without an inserted gene was also analyzed, with no 2-methylketones being detected (Figure 5). In the remaining experiment, *E. coli* C41(DE3) cells expressing SmMKS2-3 also did not produce detectable amounts of 2-methylketones. This could be possibly due to the premature truncation of SmMKS2-3 at the C-terminus resulting in the partial loss of the Hotdog domain required for catalytic activity of this enzyme.

## 4. Discussion

### 4.1. Biochemical Activities of SmMKS2s

The sequence-based gene mining discovery approach has led to the identification in eggplant, reported here, of three β-ketoacyl-ACP thioesterases. The SmMKS2-1, SmMKS2-2, and SmMKS2-3 are closely related to each other, as well as to ShMKS2, which has been demonstrated to be a plastid-targeted β-ketoacyl-ACP thioesterase [6]. When expressed in *E. coli*, SmMKS2-1 and SmMKS2-2 proteins showed thioesterase activities towards a variety of β-ketoacyl-ACPs to produce the corresponding β-ketoacids, mostly 14:1 β-ketoacid, whereas SmMKS2-3 showed no detectable activity. Chemical conversion of these compounds to methylketones during the sample treatment process for detection by GC shortens the chain by one carbon relative to the β-ketoacid (Figure 5 and Figure 6). SmMKS2-1 primarily generates 14:1 β-ketoacids, with minor β-ketoacid products ranging from 8-18 carbons in length, whereas *SmMKS2-2* has narrower specificity than SmMKS2-1, predominantly producing 12:0, 14:0 and 14:1 β-ketoacids. However, levels of extractable methylketones, which were derived from β-ketoacids, in the culture of cells expressing SmMKS2-2 were much higher than in that of cells expressing SmMKS2-1, despite the observation that the expression levels of the two proteins in *E. coli* were almost the same (Figure 6; Supplementary File 6). Despite their high sequence homology, single Hotdog fold acyl-lipid thioesterases from diverse plant species and taxa exhibit different substrate specificities and are divided into four different ALT groups based on their primary products [9]. SmMKS2-1 and SmMKS2-2 from *S. melongena* are the most similar to enzymes belonging to the Group 1 ALTs, made up of enzymes that mainly generate 14:1 β-ketoacid, such as AtALT3 from *Arabidopsis thaliana*, VvALT1 from *Vitis viniferase*, MtALT2 from *Medicago truncatula*, ZmALT2 from *Zea mays*, etc,,, However, SmMKS2-1 and SmMKS2-2 differed from the enzymes listed in this group in their minor and secondary products. *E. coli* cells expressing SmMKS2-1 or SmMKS2-2 did not produce 8:0 β-ketoacids (converted to 7:0 MK) in significant quantities, while this is one of minor products common to the Group 1 MKS2s/ALTs [9]. 

When heterologously expressed in *E. coli*, SmMKS2-3 does not exhibit thioesterase activity, as the levels of β-ketoacids in the strain harbouring SmMKS2-3 were barely above the levels that observed in negative control cells carrying the empty vector. One plausible reason for the absence of detectable β-ketoacids from the cell culture of this recombinant strain is that SmMKS2-3 has a deletion of the last 37 C-terminal amino acid residues and thus loses a part of the single Hotdog fold domain required for catalytic activity of this enzyme (Figure 1).

Based on sequence alignment, we could point out a few interesting differences between either SmMKS2-1 or SmMKS2-2 and known MKS2 sequences from other plants that might help us explore their functional differences upon expression in *E. coli*. If in vitro characterization of MKS2 remains troublesome, probing this in vivo functional difference using site-directed mutagenesis might be the best feasible analytical approach to predict whether external factors affecting substrate availability such as ACP affinity, fatty acid synthase (FAS) colocalization… or the steric constraints in the active site cavities of MKS2 enzymes govern functional differences upon expression in *E. coli*.

### 4.2. Induction of *SmMKS2-1* by Wounding, MeJA and MeSA Implies a Possible Biological Role for This Gene in Plant Defense

Plant specialized metabolites with pesticidal properties, such as medium-length 2-methylketones, confer resistance to a wide range of pests in certain crop plants [29,30]. As an example, the wild tomato species *Solanum habrochaites* ssp. *glabratum* (accession no. PI126449) is highly resistant to various anthropod pests, including spider mites (*Tetranychus urticae*), aphids (*Aphis gossypii*), glasshouse whitefly (*Trialeurodes vaporariorum*), tomato fruitworm (*Helicoverpa zea*), ten-lined potato beetle (*Leptinotarsa decimlineata*), tobacco hornworm (*Manduca sexta*), and tomato leafminer (*Tuta absoluta*), because of the high contents of 2-tridecanone and other methylketones in the leaves [30,31]. In this study, we identified at least two good candidate genes (*SmMKS2-1* and *SmMKS2-2)* for the in planta production of β-ketoacids (methylketone precursors) in eggplant, another important member of the genus *Solanum*. The SmMKS2-1 and SmMKS2-2 proteins are highly similar to the wild tomato methylketone synthase 2, ShMKS2 (67% and 56% identical) and expressing the eggplant *SmMKS2-1 or SmMKS2-2* gene in *E. coli* resulted in the production of a wider range of β-ketoacids in comparison with expressing *ShMKS2* [32] (Figure 5 and Figure 6). 

In the wild tomato species *S. habrochaites*, β-ketoacids produced by ShMKS2 are converted to methylketones for defense against pests by a β-ketoacid decarboxylase (ShMKS1) [33]. Methylketones, however, appear to be present at very low levels in other Solanaceae plant species and in *Arabidopsis thaliana* [6,7]. One possibility is that, in the plant, the β-ketofatty acids are produced and further metabolized to the other possible final products, such as secondary alcohols, or fatty acid derivatives. Another possibility is that, under normal conditions, *MKS2* and other genes putatively associated with methylketone biosynthetic pathway are expressed at low levels compared to those in the wild tomato species [32,34]. This is in line with the observation that herbivore-damaged *Arabidopsis* plants emitted more 2-pentanone (5:0 MK) than undamaged plants [35]. To assess whether the expression of *SmMKS2* genes could be triggered in *S. melongena* following stress responses, changes in their transcriptional expression in eggplant leaves after wounding or treatment with MeJA or MeSA were analyzed by qRT-PCR. Here we show that artificial wounding, MeJA treatment, or MeSA treatment of intact plants results in higher transcript levels of *SmMKS2-1*, but neither *SmMKS2-2* nor *SmMKS2-3* in leaves (Figure 4). The observation that *SmMKS2*-1 expression in leaves of treated plants was at least twofold higher compared to that in leaves of control plants at 24 h after treatment (Figure 4), combined with its broad expression pattern (Figure 3), points to a possible function of this gene in plant defense. The question remains whether this induction could lead to a detectable increase in methylketone production in planta. It is interesting to note that methylketone concentrations in the trichomes of transgenic cultivated tomato plants expressing *ShMKS2* and *ShMKS1* (or only *ShMKS2*) specifically in the trichomes were 6.5-fold higher than those found in non-transgenic control plants, however still were 100-fold lower than those found in wild tomato trichomes, suggesting that a substantial increase in methylketone production requires additional related genes such as fatty acid biosynthesis genes [6]. Extensive genetic and genomic analyses have identified additional genes associated with the high-level production of methylketones in *S. habrochaites*, such as acetyl-CoA carboxylase (ACC) and malonyl-CoA:ACP transacylase (MCAT). MKS1, ACC, and MCAT showed 355-, 2.7-, and 7.7-fold higher expression, respectively, in the trichomes of the high-MK-containing wild versus in those of low-MK-containing cultivated tomato species [32]. Therefore, it would be worth assessing the transcriptional expression of such genes, in addition to *SmMKS2* genes, following mechanical wounding or MeJA/MeSA treatment. 

### 4.3. Evolution of *SmMKS2* Genes

Plant MKS2/ALT proteins feature a highly conserved C-terminal Hotdog fold domain and an N-terminal transit peptide to direct their transport to the plastid where the acyl-ACP substrates are present [6,7]. The biological function of methylketones that are generated by the decarboxylation of β-ketoacids, products of MKS2 proteins, has been associated with plant resistance to insects [29,30]. Homologs of the eggplant SmMKS2s are found in a wide range of plant taxa and they form a distinct clade from the well characterized fatty acyl-ACP thioesterases, FatA and FatB [6,7]. FatA- and FatB-type thioesterases contain two sequential Hotdog fold domains and in a phylogenetic tree, they are grouped according to their substrate specificity, even across plant lineages [36,37]. In contrast, SmMKS2 proteins cluster mainly with other single Hotdog fold fatty acyl-ACP thioesterases from the same or related species (Figure 2). The phylogenetic analysis shown in Figure 2 indicates that a series of gene duplications occurred recently after the split between Arabidopsis and the Solanaceae species investigated here. *AtALT1*, *AtALT2*, *AtALT3* and *AtALT4* were created by such a duplication in *Arabidopsis*. Examination of the clade of *MKS2* genes from the Solanaceae species indicates that duplications and divergence of the original *MKS2* gene in this clade occurred within Solanaceae, leading to the first lineage including *ShMKS2* and *SlMKS2c* and a second gene lineage. This second gene lineage further duplicated, forming two new branches: one leading to the gene *SmMKS2-2* and the other leading to the ancestral gene of other *MKS2s* from *S. lycopersicum* (*SlMKS2a* and *SlMKS2b*), *S. melongena* (*SmMKS2-1* and *SmMKS2-3*), *S. tuberosum* (StMKS2-1), *Capsicum annuum* (*CaMKS2*), and *Nicotiana tabacum* (*NtMKS2-1* and *NtMKS2-2*).

It has been reported that there are four MKS2-like sequences in the Arabidopsis (*Arabidopsis thaliana*) and maize (*Zea mays*) genomes [7,9], three in the cultivated tomato (*Solanum lycopersicum*) genomes [6], two in the *Brachypodium distachyon* genomes [9], and one in the wild tomato (*Solanum habrochaites*), grape (*Vitis vinifera*) and *Cannabis sativa* genomes [6,9]. It is noteworthy that in plants with only one MKS2/ALT-like thioesterase, such as *S. habrochaites*, *V. vinifera* and *C. sativa*, this enzyme generates 14-carbon β-ketoacids as a main product along with a wide range of minor products [6,9] and thus might play a broad role in plant defense, while plants with multiple MKS2/ALTs such as *Arabidopsis*, cultivated tomato and eggplant might have evolved paralogs to play more specific biological roles [7]. SmMKS2-1 possesses the broad expression pattern (Figure 3) and biochemical activity, indicating that it may be involved in more than one biological function in eggplant. SmMKS2-2, however, is more restricted in both expression profiles and substrate preferences, and all products formed by SmMKS2-2 were also produced by SmMKS2-1 (Figure 5 and Figure 6). *SmMKS2-1* may have functional overlap with the *SmMKS2-2* in tissues where both genes are expressed, but it presumably also has a different role. *SmMKS2-1* is the only gene that is expressed in both aerial and root tissues (Figure 3) and upregulated by MeSA, MeJA and wounding (Figure 4), suggesting its possible role in plant defense. *SmMKS2-2* was expressed mostly in flowers where it may be involved in production of odor compounds to attract insect pollinators. Although eggplant is generally a self-pollinated crop, in some cases cross-pollination may occur in nature for long-styled flowers [38]. Fatty acid derivatives in general and the odd chain methylketones in particular are often part of floral scent mixtures attractive to visitors of certain flowers [39,40].

## 5. Conclusions

In this study, we isolated and characterized SmMKS2-1, SmMKS2-2, and SmMKS2-3 from eggplant as putative orthologs of ShMKS2 from wild tomato species. It appears that divergence of both expression profiles and biochemical activity is occurring with different members of this gene group in *S. melongena*. *SmMKS2* genes showed different patterns of expression in the plant, and in response to wounding, methyl jasmonate and methyl salicylate. When being expressed in *E. coli*, SmMKS2-1 and SmMKS2-2 were functional β-ketoacyl-ACP thioesterases, producing a wide range of β-ketoacid, mostly β-ketomyristic acid (14:1). SmMKS2-2 exhibited narrower substrate preferences but higher activity towards those substrates than SmMKS2-1. The eggplant thioesterases provide opportunities for investigating their products in the crop plant, and their contribution to insect resistance, fragrance and flavor formation, as well as the production of biofuels and renewable chemicals. This finding further highlights how a homology-based gene mining approach in plants can reveal chemical diversity of natural products that might not be exposed by traditional approaches.

## Supplementary Materials

The following are available online at https://www.mdpi.com/2073-4425/10/7/549/s1, Supplementary File 1: PCR amplification of *SmMKS2-1*, *SmMKS2-2*, and *SmMKS2-3* using cDNA template prepared from stems (*SmMKS2-1* and *SmMKS2-3)* and flowers (*SmMKS2-2)* of *S. melongena* and gene-specific primers was checked by agarose gel electrophoresis. Lane S: PCR using cDNA template; lane C: PCR without using cDNA template (negative control); laneM: DNA ladder. Supplementary File 2: Nucleotide sequence of SmMKS2-1 and the amino acid sequence of the protein it encodes. Exon sequences are bold with the amino acids sequence above the codons. Supplementary File 3: Nucleotide sequence of SmMKS2-2 and the amino acid sequence of the protein it encodes. Exon sequences are bold with the amino acids sequence above the codons. Supplementary File 4: Nucleotide sequence of SmMKS2-3 and the amino acid sequence of the protein it encodes. Exon sequences are bold with the amino acids sequence above the codons. Underlined sequence corresponds to a 47-insertion in the coding region that causes a frameshift. Supplementary File 5: Evolutionary relationships based on Bayesian inference of the Solanaceae *MKS2*s and previously reported *ALT*s from *A. thaliana*. An HKY85 model was utilized with 4 γ rate variation categories (GR + G). The chain length was 1,100,000 (4 heated chains with a length of 0.2), and trees were sampled every 200 trees. Branch lengths were unconstrained. Supplementary File 6: Analyses of the recombinant SmMKS2 proteins expressed in the *E. coli* C41(DE3) strain. Both intracellular soluble (S) and insoluble (I) protein fractions were loaded on sodium dodecyl sulfate polyacrylamide gel electrophoresis (SDS–PAGE) gel, and proteins were stained with Coomassie Brilliant Blue R250. From the left to right: Lanes 1–2 stand for protein fractions prepared from cells transformed with empty vector as a negative control. Lanes 3–4, 6–7 and 7–9 show the expression of SmMKS2-1, SmMKS2-3, and SmMKS2-3 in *E. coli* C41(DE3) cells, respectively. Lane 5 indicates the molecular weight standards and the arrows indicate the target proteins. Supplementary File 7: Mass spectrometry analysis of 2-pentadecanone and monounsaturated methylketones in Figure 5. Chromatograms are shown in Figure 5 in the text. Supplementary File 8: Synthetic oligonucleotide used in this study. Restriction sites on primers are underlined and primer overhangs are in bold. Start codons are in blue and stop codons in red. “Truncated” refers to the open reading frame (ORF) without the transit peptide coding region. A brief description of what each primer was used for is provided.
