# Supplementary material for: Characterization of Solanum melongena Thioesterases Related to Tomato Methylketone Synthase 2
Source: Genes (Basel). 2019 Jul 18;10(7):549. doi: 10.3390/genes10070549 (PMC6678348; doi:10.3390/genes10070549)
Supplement: Supplementary file 1 [file genes-10-00549-s001.zip › Revised supplemental files/Supplemental file 8.pdf]

Supplemental Table 1. Synthetic oligonucleotide used in this study

Restriction sites on primers are underlined and primer overhangs are in bold. Start codons are in blue and stop codons in red. “Truncated” refers to the ORF without the transit peptide coding region. A brief description of what each primer was used for is provided.

**Primers for isolation and cloning of full-length SmMKS2 cDNAs**		
Primer name	Primer sequence (5’→3’)	Brief description
SmMKS2-1-F	**CCATGG**ATGTCACAATCCCTAGGA	5' primer for full-length *SmMKS2-1*
SmMKS2-1-f/t-R	**CTCGAG**TTAGTTAGATGCCTTCTGG	3' primer for full-length *SmMKS2*-1
SmMKS2-2-F	CCAATGTTTCCAATCTAGTAGC	5' primer for full-length *SmMKS2-2*
SmMKS2-2-f/t-R	**CTCGAG**TTACTTCAATGCCTCCTG	3' primer for full-length *SmMKS2-2*
SmMKS2-3-F	**AAGCTTA**ATGTCTCGGTCCCCTTTG	5' primer for full-length *SmMKS2-3*
SmMKS2-3-R	**GGATCCG**ACTAATTTTGATCTGATTTC TGAC	3' primer for full-length *SmMKS2-3*
**Primers for heterologous protein expression in *E. coli***		
SmMKS2-1-t-F	**CAT**ATGGGTGAGTTTCATGA	5' primer for truncated *SmMKS2-1*
SmMKS2-1-f/t-R	**CTCGAG**TTAGTTAGATGCCTTCTGG	3' primer for truncated *SmMKS2-1*
SmMKS2-2-t-F	**CAT**ATGGGTCAGTTCTATGAAA	5' primer for truncated *SmMKS2-2*
SmMKS2-2-f/t-R	**CTCGAG**TTACTTCAATGCCTCCTG	3' primer for truncated *SmMKS2-2*
SmMKS2-3-t-F	**CAT**ATGGGTGAGTTTCATGAAGTTG	5' primer for truncated *SmMKS2-3*

SmMKS2-3-t-R	**<u>CTCGAG</u>**TCACGACGAACCTATCTCC	3' primer for truncated *SmMKS2-3*
**qRT-PCR primers for real-time PCR analysis**		
APRT-qF	TGGTTGCAGGTGTGGAAG	5' primer for *APRT* qRT-PCR
APRT-qR	GGTAGCTTCTTAGGTTTCCTC	5' primer for *APRT* qRT-PCR
SmMKS2-1-qF	CCAGATCAAGAGCCAATC	5' primer for *SmMKS2-1* qRT-PCR
SmMKS2-1-qR	AATTTCGATCTGAACTCTGG	3' primer for *SmMKS2-1* qRT-PCR
SmMKS2-2-qF	CTTCCAAATCAAGAGCCTG	5' primer for *SmMKS2-2* qRT-PCR
SmMKS2-2-qR	GCAATTTTAATTTGACCTCTGA	3' primer for *SmMKS2-2* qRT-PCR
SmMKS2-3-qF	ATCCCTCTCCGGCAACTC	5' primer for *SmMKS2-3* qRT-PCR
SmMKS2-3-qR	CATGAAACTCACCCATTCTTTG	3' primer for *SmMKS2-3* qRT-PCR
**Primers for isolation of genomic sequences**		
gSmMKS2-1-F1	**<u>CCATGG</u>**ATGTCACAATCCCTAGGA	5' primer for partial *SmMKS2-1* (1st fragment)
gSmMKS2-1-R1	GGAGTAACTAGCAAGATCTGA	3' primer for partial *SmMKS2-1* (1st fragment)
gSmMKS2-1-F2	CCAGTTTCCAATGTAATAAGC	5' primer for partial *SmMKS2-1* (2nd fragment)
gSmMKS2-1-R2	GGAGCTAGATACTTAAGTGACA	3' primer for partial *SmMKS2-1* (2nd fragment)
gSmMKS2-1-F3	CATAAGTGCTGATGAAGTG	5' primer for partial *SmMKS2-1* (3rd fragment)

gSmMKS2-1-R3	**<u>CTCGAG</u>**TTAGTTAGATGCCTTCTGG	3' primer for partial *SmMKS2-1* (3rd fragment)
gSmMKS2-2-F1	CCAATGTTTCCAATCTAGTAGC	5' primer for partial *SmMKS2-2* (1st fragment)
gSmMKS2-2-R1	CTTACGAGATCACCTTAAGACAC	3' primer for partial *SmMKS2-2* (1st fragment)
gSmMKS2-2-F2	ACAATGTTAAGCTAGAGTGCC	5' primer for partial *SmMKS2-2* (2nd fragment)
gSmMKS2-2-R2	GCGTACTACTTCATCAGCAC	3' primer for partial *SmMKS2-2* (2nd fragment)
gSmMKS2-3-F3	ATGGCTTACATTTCTCTCC	5' primer for partial *SmMKS2-1* (3rd fragment)
gSmMKS2-3-R3	ATGAACAAAGCTCTTCTGG	3' primer for partial *SmMKS2-1* (3rd fragment)
gSmMKS2-3-F1	ATGTCTCGGTCCCCTTTGATTG	5' primer for partial *SmMKS2-3* (1st fragment)
gSmMKS2-3-R1	CGAGGATCTTAATGATGTACATGC	3' primer for partial *SmMKS2-3* (1st fragment)
gSmMKS2-3-F2	GGCATAAGTGTTGATGAAGTGG	5' primer for partial *SmMKS2-3* (2nd fragment)
gSmMKS2-3-R2	CCAAGCGATAATAATGGAGGTAAC	3' primer for partial *SmMKS2-3* (2nd fragment)
