# Supplementary figures and images for: Characterization of Solanum melongena Thioesterases Related to Tomato Methylketone Synthase 2
Source: Genes (Basel). 2019 Jul 18;10(7):549. doi: 10.3390/genes10070549 (PMC6678348; doi:10.3390/genes10070549)

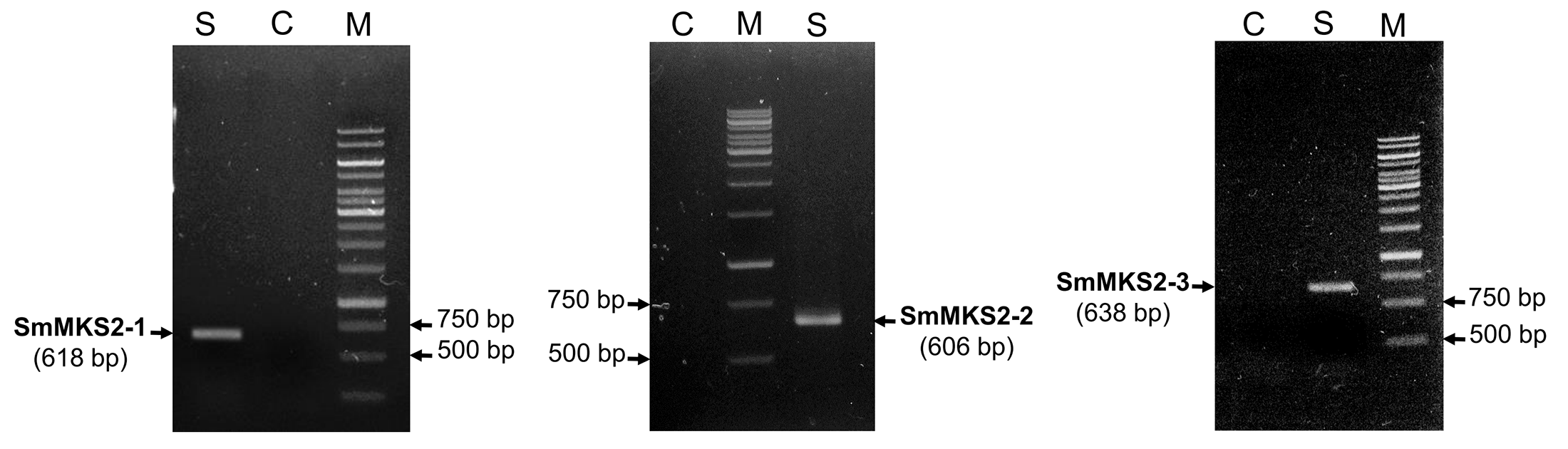

Supplement: Supplementary file 1 [file genes-10-00549-s001.zip › Revised supplemental files/Supplemental file 1.tif]

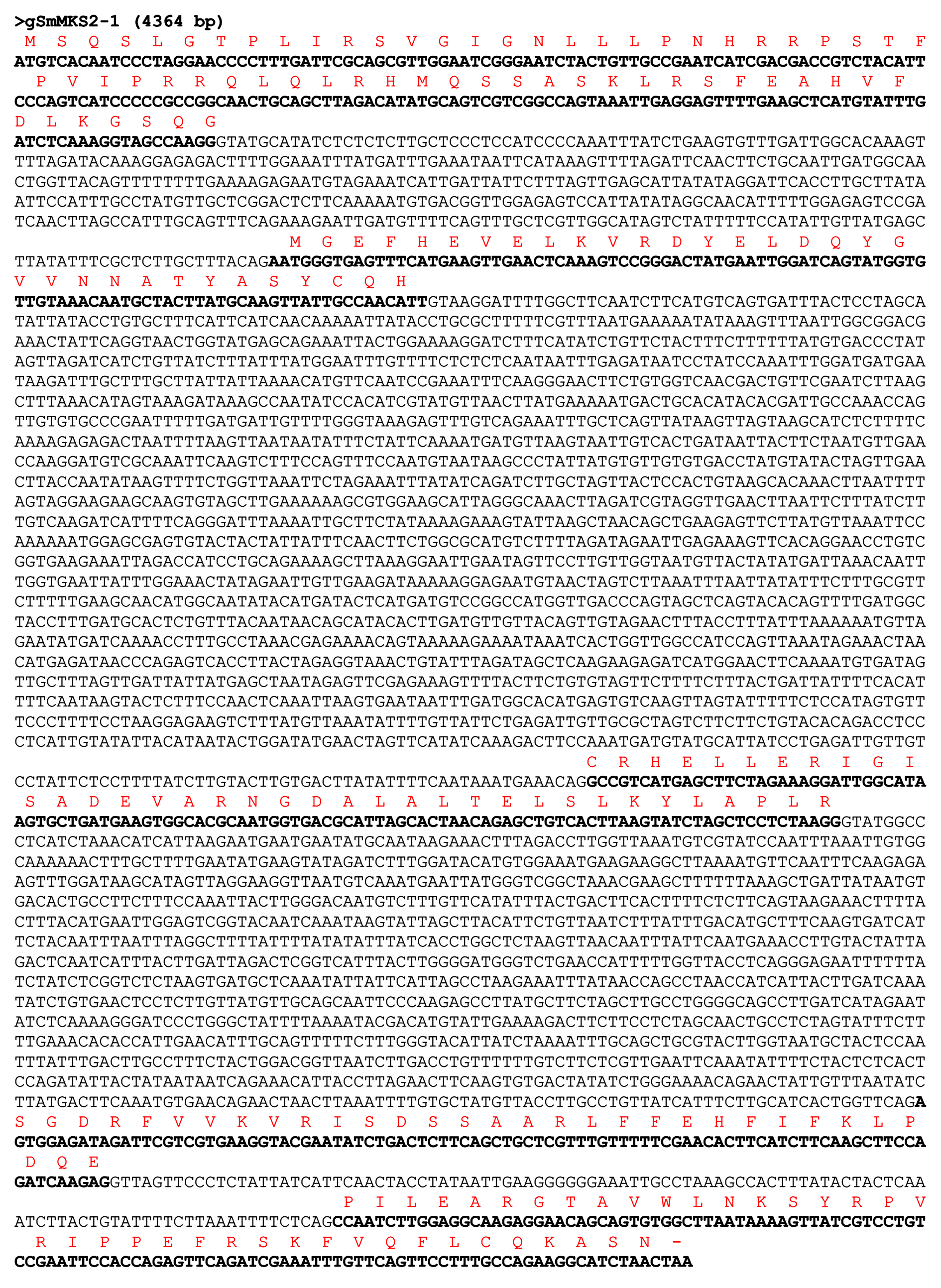

Supplement: Supplementary file 1 [file genes-10-00549-s001.zip › Revised supplemental files/Supplemental file 2.tif]

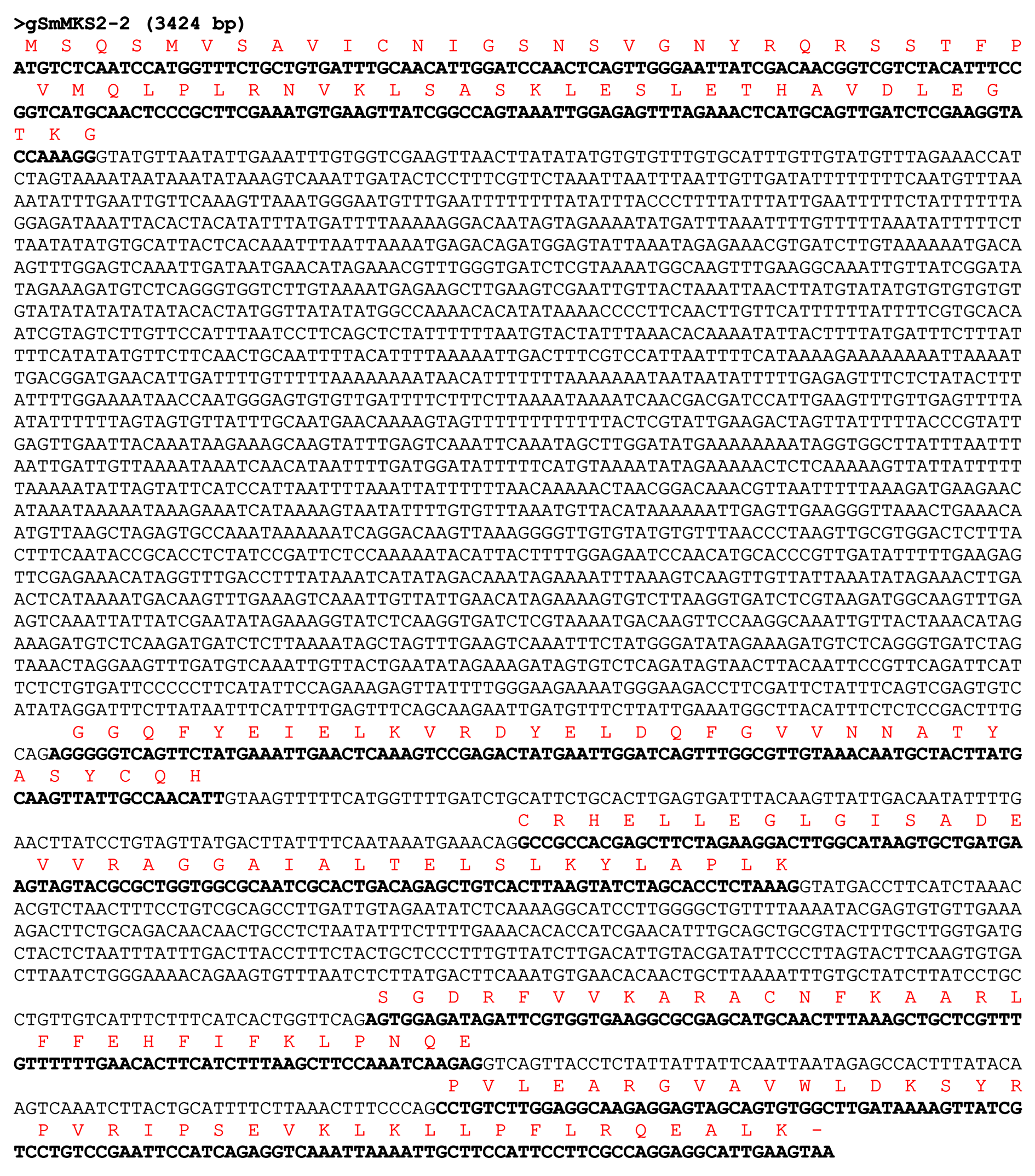

Supplement: Supplementary file 1 [file genes-10-00549-s001.zip › Revised supplemental files/Supplemental file 3.tif]

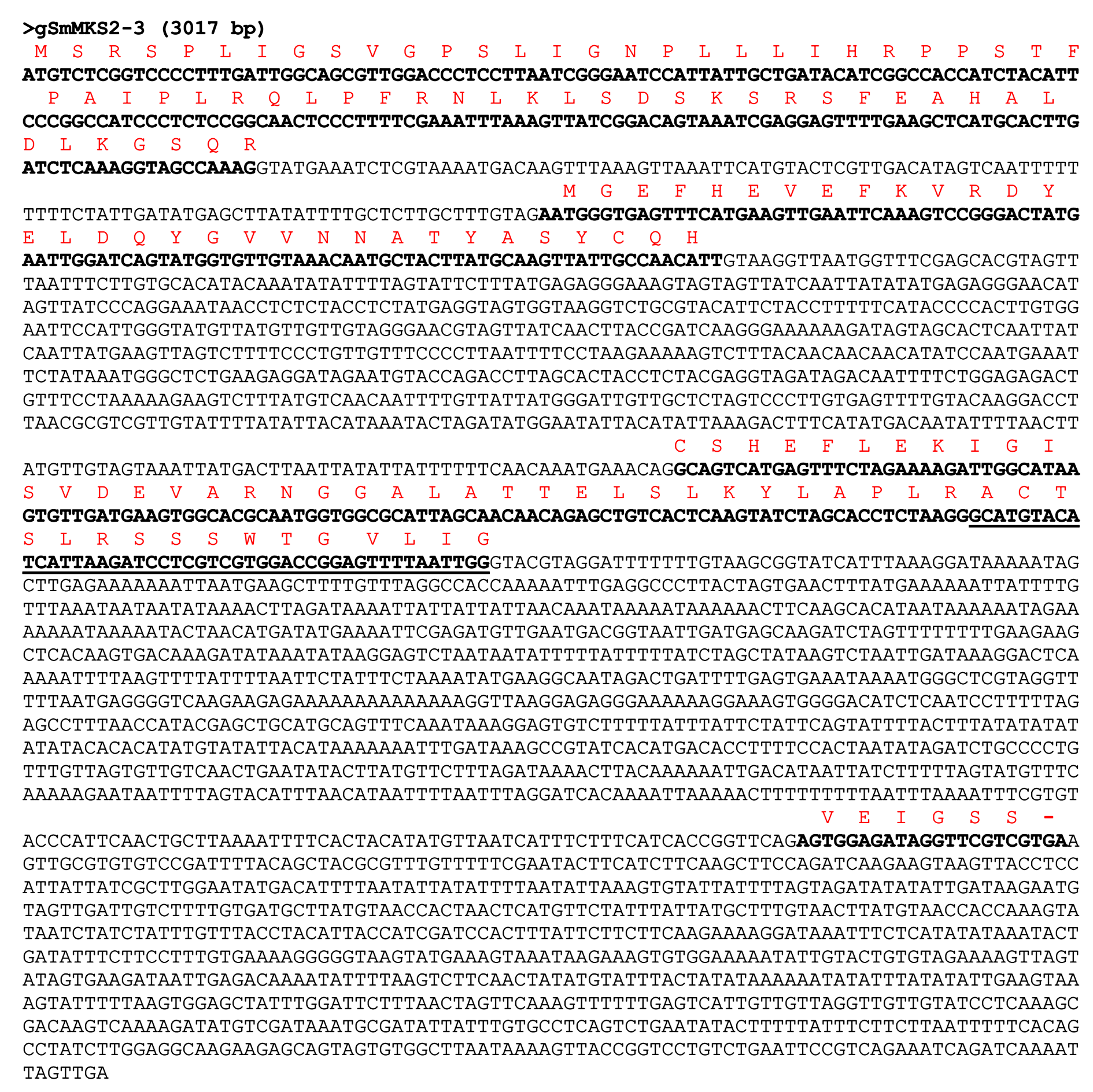

Supplement: Supplementary file 1 [file genes-10-00549-s001.zip › Revised supplemental files/Supplemental file 4.tif]

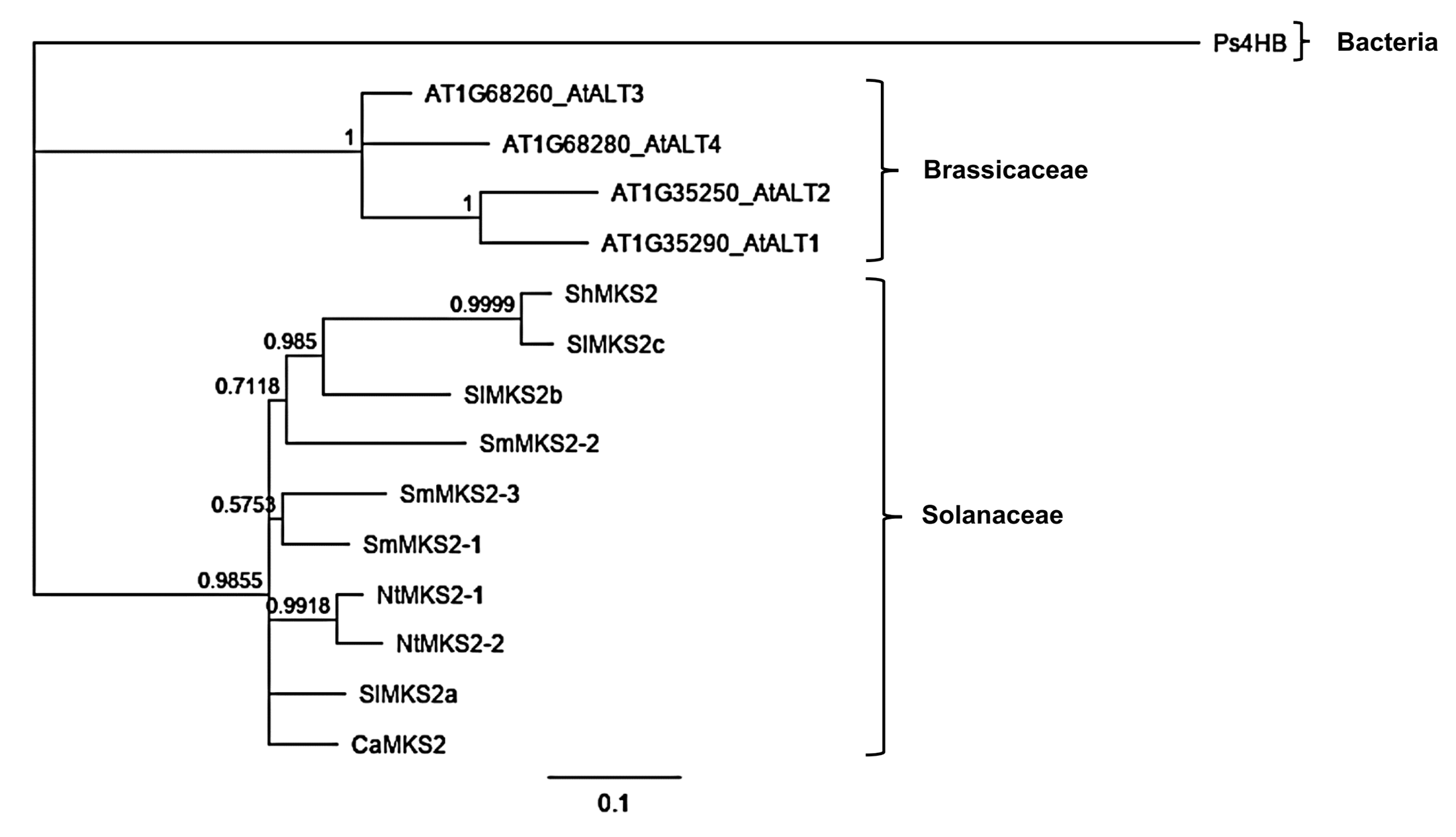

Supplement: Supplementary file 1 [file genes-10-00549-s001.zip › Revised supplemental files/Supplemental file 5.tif]

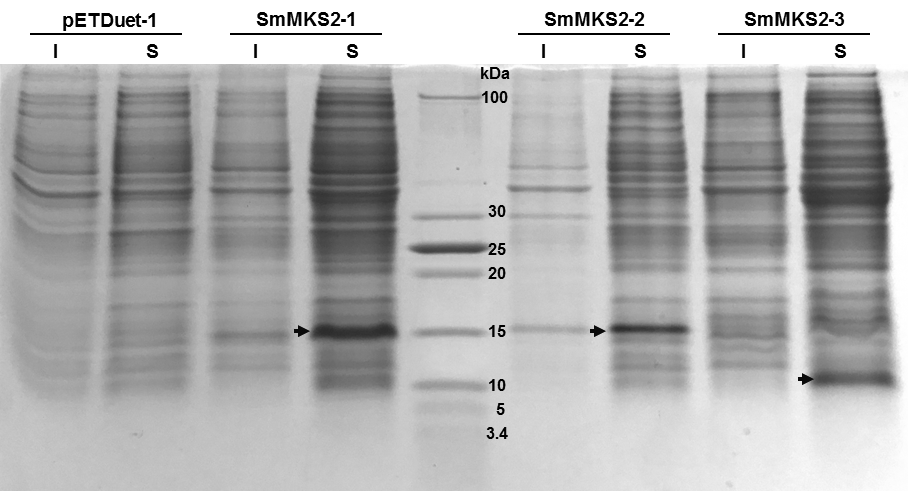

Supplement: Supplementary file 1 [file genes-10-00549-s001.zip › Revised supplemental files/Supplemental file 6.tif]

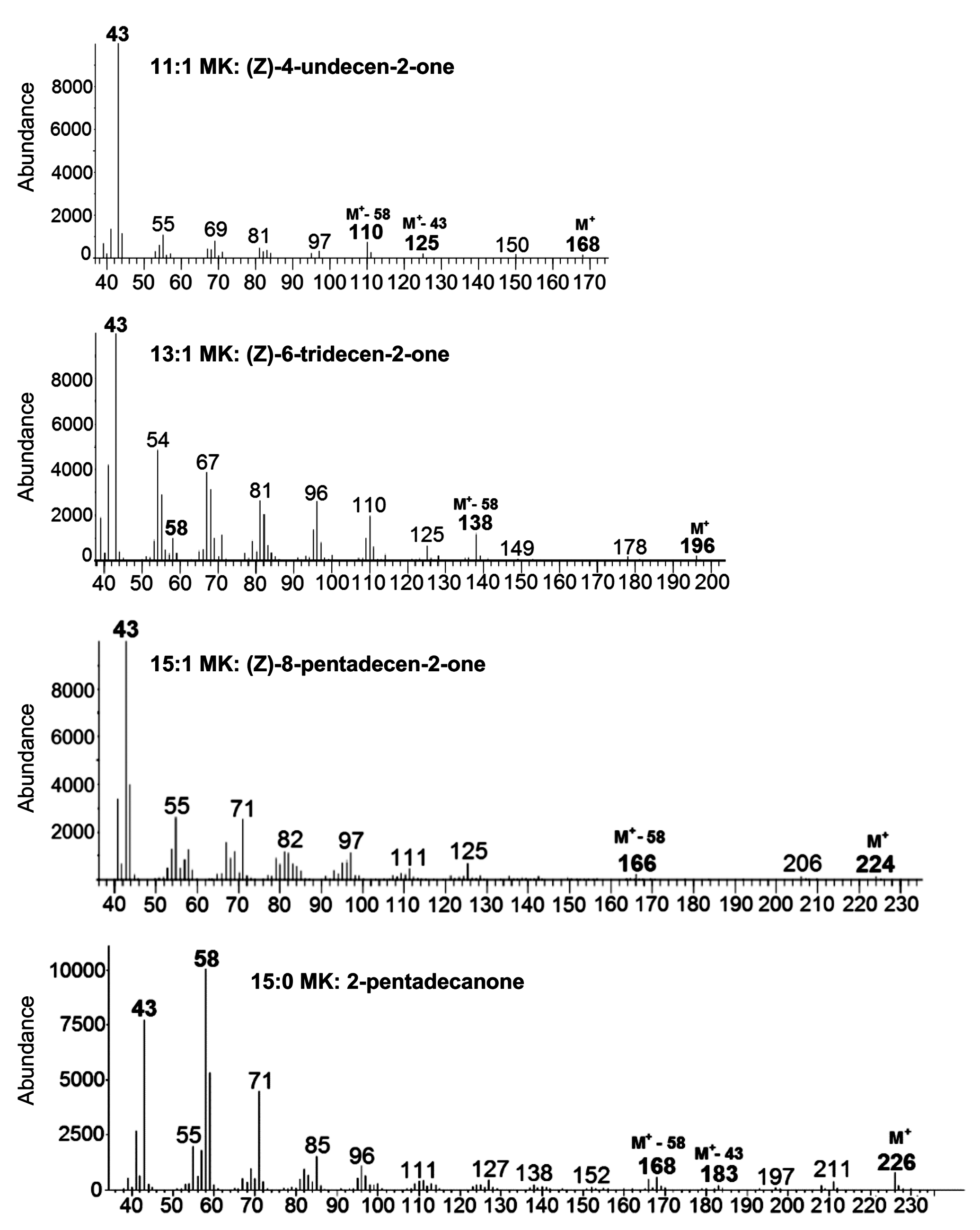

Supplement: Supplementary file 1 [file genes-10-00549-s001.zip › Revised supplemental files/Supplemental file 7.tif]
